# The Effects of Temperature and Pressure on Protein-Ligand Binding in the Presence of Mars-Relevant Salts †

**DOI:** 10.3390/biology10070687

**Published:** 2021-07-20

**Authors:** Nisrine Jahmidi-Azizi, Rosario Oliva, Stewart Gault, Charles S. Cockell, Roland Winter

**Affiliations:** 1 Physical Chemistry I—Biophysical Chemistry, Department of Chemistry and Chemical Biology, TU Dortmund University, Otto-Hahn Street 4a, 44227 Dortmund, Germany; nisrine.jahmidi@tu-dortmund.de; 2 UK Centre for Astrobiology, SUPA School of Physics and Astronomy, University of Edinburgh, James Clerk Maxwell Building, Peter Guthrie Tait Road, Edinburgh EH9 3FD, UK; s.a.gault@sms.ed.ac.uk (S.G.); c.s.cockell@ed.ac.uk (C.S.C.)

**Keywords:** protein–ligand binding, high pressure, Martian salts, perchlorate, BSA, ANS

## Abstract

**Simple Summary:**

Interactions of ligands with proteins are central to all reactions in the biological cell. How such reactions are affected by harsh environmental conditions, such as low temperatures, high pressures, and high concentrations of biologically destructive salts, is still largely unknown. Our work focused on specific salts found on Mars to understand whether the planet’s potentially liquid, water-rich subsurface harbors conditions that are theoretically favorable for life. Our data show that, while magnesium chloride and sulfate do not significantly alter protein–ligand interactions, the perchlorate ion strongly affects protein–ligand binding. However, the temperature and pressure conditions encountered on Mars do not necessarily preclude protein–ligand interactions of the type studied here.

**Abstract:**

Protein–ligand interactions are fundamental to all biochemical processes. Generally, these processes are studied at ambient temperature and pressure conditions. We investigated the binding of the small ligand 8-anilinonaphthalene-1-sulfonic acid (ANS) to the multifunctional protein bovine serum albumin (BSA) at ambient and low temperatures and at high pressure conditions, in the presence of ions associated with the surface and subsurface of Mars, including the chaotropic perchlorate ion. We found that salts such as magnesium chloride and sulfate only slightly affect the protein–ligand complex formation. In contrast, magnesium perchlorate strongly affects the interaction between ANS and BSA at the single site level, leading to a change in stoichiometry and strength of ligand binding. Interestingly, both a decrease in temperature and an increase in pressure favor the ligand binding process, resulting in a negative change in protein–ligand binding volume. This suggests that biochemical reactions that are fundamental for the regulation of biological processes are theoretically possible outside standard temperature and pressure conditions, such as in the harsh conditions of the Martian subsurface.

## 1. Introduction

Protein–ligand recognition and binding are fundamental to all biochemical processes and are essential for all life forms [1,2,3,4,5]. Hence, elucidating the nature and strength of the driving forces involved in the ligand binding processes is of particular interest in the biosciences. In most cases, non-covalent bonds, such as electrostatic and hydrophobic interactions, ensure formation of the protein–ligand complexes [5]. In a molecular picture, protein–ligand interactions may not strictly follow a simple binding process; instead, they may be accompanied by conformational as well as hydration changes of the protein and potentially also the ligand. Owing to the inherent complexity of the process, many aspects of ligand binding have not been fully explored, yet. This is particularly true for complex solution conditions, such as ligand binding in cellulo or in the presence of high concentrations of co-solutes or at extreme environmental conditions, such as at low/high temperatures and high hydrostatic pressures (HHP). HHP environments are, for example, encountered in the deep sea or in subsurface environments where pressures up to the kbar regime are encountered (1 kbar = 100 MPa). Hence, knowledge about high hydrostatic pressure effects on biological systems is also fundamental for our understanding of life being exposed to such harsh conditions and of the physical limits of life in general [6,7,8,9,10].

Of interest is to explore the theoretical capacity of other planetary bodies, such as Mars, to support life. Mars is known to have hosted a vigorous hydrological regime in its early history. Although we do not know if Mars ever hosted life, we could ask the question whether conditions on that planet could theoretically support similar biological processes observed on Earth and therefore whether Mars or Mars-like geological conditions on any planet would act as a barrier to life as we know it. In particular, as water was lost from Mars, brines may have formed. Today, the planet hosts localized high concentrations of salts such as chlorides [11], sulfates [12], and perchlorates [13]. Although the surface of Mars is today at the triple point, the subsurface may still host liquid water as it did in the past [14,15,16]. These chemical constituents of the planetary environment raise the question of how they, in combination with high pressures associated with the subsurface, would influence the theoretical habitability of that planet.

In this work, we set out to investigate the effect of temperature, pressure, and Mars-relevant salts, including MgCl_2_, Mg(ClO_4_)_2_, and MgSO_4_, on the binding characteristics of the small aromatic ligand 8-anilinonaphthalene-1-sulfonic acid (ANS) to the multifunctional protein bovine serum albumin (BSA). To this end, pressure-dependent fluorescence spectroscopy was applied, supplemented by circular dichroism (CD) experiments.

## 2. Materials and Methods

### 2.1. Materials

The protein bovine serum albumin (BSA, molecular weight of 66 kDa, 583 residues) in the form of lyophilized powder, the fluorophore 8-anilinonaphthalene-1-sulfonic acid (ANS), the salts MgCl_2_, Mg(ClO_4_)_2_ and MgSO_4_ were all purchased from Sigma Aldrich Chemicals (Taufkirchen, Germany). All the sample solutions were prepared in the pressure stable 10 mM Tris-HCl buffer, at the pH of 7.4. Deionized water was used for all buffer and sample preparations.

### 2.2. Samples Preparation

The stock solution of the protein BSA was prepared by dissolving the lyophilized powder in Tris-HCl buffer. The exact concentration was determined by measuring the absorbance at 280 nm with a UV-1800 spectrometer from Shimadzu Corporation (Kyoto, Japan), using a molar extinction coefficient of 43600 M^−1^ cm^−1^ [17]. A stock solution of the fluorophore ANS was prepared by dissolving it in water. After dilution in buffer, the exact concentration was determined by measuring the absorbance, using a molar extinction coefficient of *ε* (350 nm) = 4950 M^−1^ cm^−1^ [17]. All the samples were prepared by diluting BSA and ANS in 10 mM Tris-HCl buffer, pH 7.4, in the absence and presence of 250 mM MgCl_2_, Mg(ClO_4_)_2_ or MgSO_4_.

### 2.3. Steady-State Fluorescence Spectroscopy

The extent of complex formation between ANS and BSA was followed by means of steady-state fluorescence spectroscopy using a K2 fluorometer from ISS, Inc. (Champaign, IL, USA). The binding isotherms were obtained by recording ANS emission spectra by exciting the solutions at 350 nm and recording the emission intensities in the range 400–550 nm. The width slits of the excitation and emission monochromators were both set to 8 nm. Briefly, a series of solutions with a fixed concentration of ANS at ~5 µM were prepared, and the concentration of BSA was varied between 0 and ~40 µM. Then estimation of the binding constants (*K*_b_) was performed by using a plot of Δ*F*  =  *F* − *F*_0_ (where *F* is the fluorescence intensity of ANS at the maximum in the presence of BSA, and *F*_0_ is the intensity of ANS in the absence of BSA) as a function of total BSA concentration and fitting the experimental data according to an equivalent and independent binding sites model or to two classes of non-equivalent and independent binding-site models, as described in detail in Reference [17].

For the pressure dependent measurements, the high-pressure cell system from ISS and quartz cuvettes were used. The pressure was controlled by means of a manual pump, and water was used as pressurizing fluid. A pressure range from 1 to 2000 bar was explored. The ANS and BSA solutions were mixed, vortexed, and then filled into the sample cell, which was sealed with DuraSeal^TM^ laboratory stretch film and placed into the high-pressure vessel. The stretch film allowed for the pressure transmission in the sample cuvette.

### 2.4. Circular Dichroism Spectroscopy

Circular dichroism (CD) spectroscopy experiments were performed in the Far-UV region (190–260 nm) in order to determine the secondary structure of the protein BSA in the presence of 250 mM of MgCl_2_, Mg(ClO_4_)_2_, or MgSO_4_ and at the temperatures of 5, 15, and 25 °C. CD spectra of a 14 µM BSA solution were recorded by using a 0.01 cm path-length quartz by means of a Jasco J-715 spectropolarimeter (Jasco Corporation, Tokyo, Japan). The instrument parameters were set as follows: scan rate of 50 nm min^−1^, response of 2 s, and bandwidth of 4 nm. For each sample, a background blank (neat buffer or salts-containing buffer) was subtracted. The recorded spectra are the results of three accumulations, and they were normalized per mole of residue.

## 3. Results

In a previous study it was shown that the small aromatic ligand ANS is able to bind to the protein BSA with a binding constant, *K*_b_, of the order of 10^6^ M^−1^ in 10 mM Tris-HCl buffer (neat buffer conditions) at ambient temperature, i.e., 25 °C [11]. It was also demonstrated that three ANS molecules are bound on average to one BSA molecule and that the binding process can be well described by assuming that all the three ANS molecules can interact with the same affinity [17]. In order to investigate the impact of the Mars-relevant salts on the complex formation between ANS and BSA, we performed a series of fluorescence spectroscopic experiments. By varying the temperature and pressure, a temperature range from 5 to 25 °C and a pressure range from ambient pressure (1 bar) to 2000 bar was covered.

**Figure 1:**
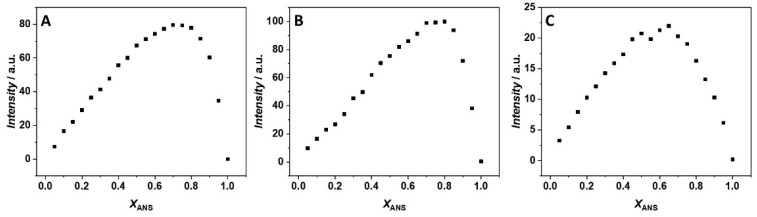
Job’s plot for ANS/BSA complex formation obtained at the temperature of 25 °C and pressure of 1 bar in the presence of 250 mM (**A**) MgCl_2_, (**B**) MgSO_4_, and (**C**) Mg(ClO_4_)_2._ The total concentration ([ANS] + [BSA]) was 35 µM. The fluorescence intensity is reported in arbitrary units (a.u.).

First, we explored the impact of 250 mM MgCl_2_, Mg(ClO_4_)_2_, and MgSO_4_ on the ligand binding process at 25 °C and ambient pressure (1 bar). To be able to determine the binding constant, it is of fundamental importance to exactly know the stoichiometry of the complex formed. In this way, a proper binding model can be applied in the data analysis of the measured binding isotherms, providing the most appropriate value of the binding constant, *K*_b_. We applied the method of continuous variation (or Job’s plot) for the estimation of the binding stoichiometry [18,19,20] by means of steady-state fluorescence spectroscopy. A series of solutions were prepared such that the total concentration (i.e., [ANS] + [BSA]) is constant, while the mole fractions of the interacting partners were varied. Discontinuities in the plot of fluorescence intensity vs. *x*_ANS_ (where *x*_ANS_ is the mole fraction of ANS) are indicative of the stoichiometry of the complex formed. Figure 1 shows the Job’s plot obtained in the presence of the three salts at *T* = 25 °C and *p* = 1 bar.

An inspection of Figure 1A reveals the presence of one inflection point in the presence of MgCl_2_ which is centered at *x*_ANS_ ≈ 0.75, indicating the formation of a 1:3 (BSA:ANS) complex. Qualitatively, the same result was also obtained in the MgSO_4_ containing solution: the presence of one inflection point at *x*_ANS_ ≈ 0.75 suggests that, again, three ANS molecules are bound to one BSA molecule (Figure 1B). The same stoichiometry was previously observed in the same buffer but in the absence of any salt [17], suggesting that both magnesium chloride and sulfate have no significant impact on the stoichiometry of the BSA:ANS complex.

A completely different scenario was observed in the presence of Mg(ClO_4_)_2_. The Job’s plot is characterized by the presence of two distinct inflection points (Figure 1C). One is centered at *x*_ANS_ = 0.5, and the other one at *x*_ANS_ ≈ 0.65, suggesting that only two ANS molecules are bound to one BSA molecule. The presence of two distinct inflection points is indicative of a non-equivalence of binding sites; otherwise, only one inflection point should be observed, as in the case of the MgCl_2_ and MgSO_4_ solutions. Thus, Mg(ClO_4_)_2_ is able to strongly modulate the formation of the complex at the level of single binding sites, inhibiting the binding to one site and leading to the loss of equivalence of the two remaining sites.

Then, we explored the effect of temperature on the stoichiometry of the ANS:BSA complex. We determined the Job’s plots also at 5 and 15 °C. The Job’s plot data obtained in the presence of MgCl_2_, MgSO_4_, and Mg(ClO_4_)_2_ are reported in Supplementary Materials Figures S1–S3, respectively. An inspection of Supplementary Materials Figures S1–S3 reveals that a decrease in temperature to 5 °C does not alter the stoichiometry of the protein–ligand complex. As in the case at *T* = 25 °C, stoichiometries of 3:1, 3:1, and 2:1 ANS:BSA were observed for the complex formation in the presence of MgCl_2_, MgSO_4_, and Mg(ClO_4_)_2_, respectively (Table 1).

**Figure 2:**
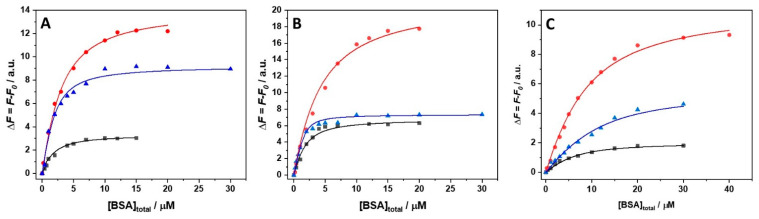
Binding isotherms for complex formation between the bovine serum albumin (BSA) and 8-anilinonaphthalene-1-sulfonic acid (ANS) obtained at the temperatures of 5 °C (black squares), 15 °C (red circles), and 25 °C (blue triangles) at *p* = 1 bar in the presence of 250 mM (**A**) MgCl_2_, (**B**) MgSO_4_, and (**C**) Mg(ClO_4_)_2_. The solid lines represent the best fit of experimental data according to an equivalent and independent binding sites model for the experiments performed in the presence of MgCl_2_ and MgSO_4_, and to two classes of independent binding-sites models for the experiments carried out in the presence of Mg(ClO_4_)_2_. The binding isotherms were obtained by plotting Δ*F* = *F* − *F*_0_ (where *F* is the fluorescence intensity of ANS at the maximum in the presence of BSA, and *F*_0_ is the intensity of ANS in the absence of BSA) as a function of total BSA concentration.

Once the stoichiometries of the complex in the presence of the salts and at the three different temperatures are known, it is possible to estimate the binding constants, *K*_b_, performing titration experiments by means of steady-state fluorescence spectroscopy. In these experiments, a solution of ANS was titrated with a BSA solution. The ANS is characterized by a very low quantum yield when it is free in solution [21]. Instead, when the aromatic ligand is bound to the protein’s hydrophobic pockets, a strong increase of the fluorescence intensity is observed. Thus, the extent of binding can be readily determined by following the fluorescence increase of ANS. The subsequent data analysis was performed according to the stoichiometries inferred by the Job’s plot experiments. In MgCl_2_ and MgSO_4_ containing buffer with one class of site only, the experimental points of the titration experiment were fitted according to an equivalent and independent binding sites model. Instead, in the presence of Mg(ClO_4_)_2_, two distinct binding modes were observed; hence, a two-classes-of-independent-binding-sites model was used. Figure 2A–C depict the binding isotherms at ambient pressure, obtained in the presence of 250 mM MgCl_2_, MgSO_4_, and Mg(ClO_4_)_2_ at 5, 15, and 25 °C. The values of the binding constants determined are collected in Table 1.

In the presence of MgCl_2_, decreasing the temperature from 25 to 15 °C, a prominent decrease of *K*_b_ (~90%) was observed. At 5 °C, the *K*_b_-value is only slightly higher compared to the value at 15 °C, however. Thus, the system exhibits a complex non-linear temperature dependence of the overall binding constant measured. A very similar behavior was also observed in the presence of MgSO_4_, i.e., the decrease of the temperature disfavors formation of the complex, but in a non-linear way. In the presence of Mg(ClO_4_)_2_, the *K*_b_-values decrease as well upon lowering the temperature. The decrease of *K*_b1_ seems to be less pronounced with respect to the decrease of *K*_b2_, indicating that the two different binding sites are characterized by different binding energetics, which is probably due to the differences in the non-covalent interactions established upon binding. It is important to note that decreasing the temperature leads to similar values of the two binding constants.

**Figure 3:**
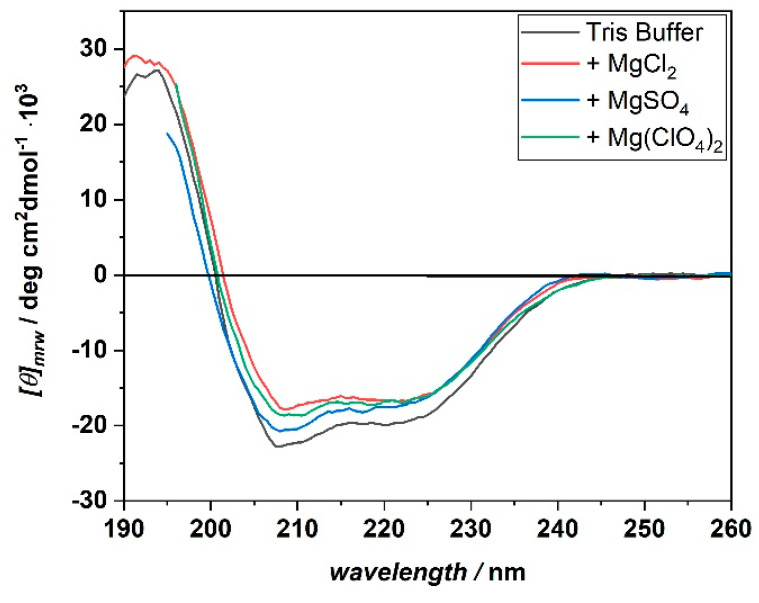
Far-UV CD spectra of BSA in the presence of 250 mM MgCl_2_ (red spectrum), MgSO_4_ (blue spectrum), and Mg(ClO_4_)_2_ (green spectrum). For reference, the CD spectrum of BSA in neat buffer condition (black spectrum) is also reported. All the spectra were acquired at the temperature of 25 °C, in 10 mM Tris-HCl buffer, pH 7.4. The CD spectra are reported by plotting the molar ellipticity ([*θ*]_mrw_) as a function of the wavelength, in nm.

Principally, the differences observed for the effect of temperature and salt on complex formation could be ascribed to conformational changes of the protein structure. To test this possibility, circular dichroism (CD) spectra of BSA in the far-UV range were recorded. In this spectral region, information about the secondary structure of the protein can be obtained [22,23]. The spectra were acquired at temperatures of 5, 15, and 25 °C, and in the presence of the three different salts. Figure 3 shows the CD spectra of BSA recorded at 25 °C in the presence of 250 mM MgCl_2_, Mg(ClO_4_)_2_, and MgSO_4_. For reference, the CD spectrum of BSA in neat buffer (10 mM Tris-HCl, pH 7.4) is also shown.

The CD spectrum of BSA at neat buffer conditions is characterized by the presence of two minima located at about 208 and 222 nm. In addition, a positive band centered at around 195 nm is present. These spectral features are characteristic of a protein which adopts an α-helical conformation, which is in agreement with its reported structure [24]. In the salt-containing media, the CD spectra of BSA show the same general features as in the neat buffer case, signifying that even in the presence of high concentrations of these Mars-relevant salts, the protein’s secondary structure seems to be retained. The same result was obtained for the measurements carried out at the temperatures of 5 and 15 °C (Supplementary Materials Figure S4). Thus, the differences observed in the binding affinities and stoichiometries do not seem to be related to a conformational change of the protein structure. However, small and localized changes cannot be excluded a priori.

**Figure 4:**
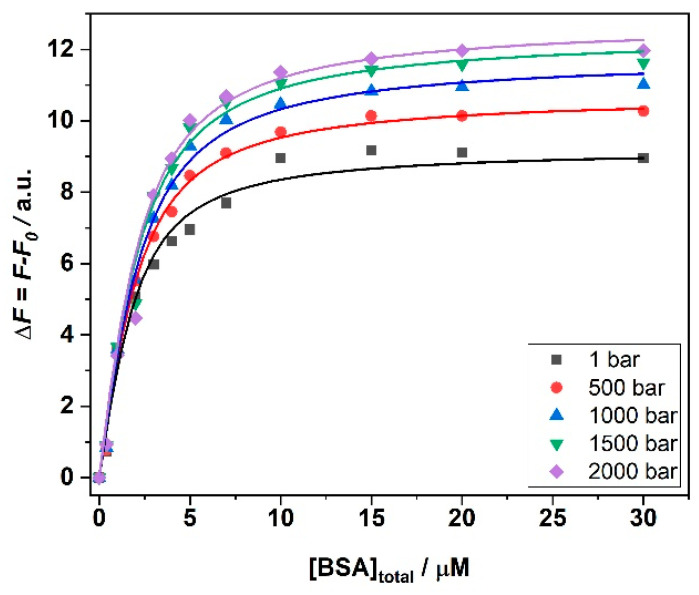
Binding isotherms for ANS/BSA complex formation obtained at *T* = 25 °C in the presence of 250 mM MgCl_2_, at the indicated pressure values. The solid lines represent the best fit of experimental data according to an equivalent and independent binding sites model. All the experiments were performed in 10 mM Tris-HCl buffer. The binding isotherms were obtained by plotting Δ*F* = *F* − *F*_0_ (where *F* is the fluorescence intensity of ANS at the maximum in the presence of BSA, and *F*_0_ is the intensity of ANS in the absence of BSA) as a function of the total BSA concentration.

In the next step, we explored the impact of high hydrostatic pressure (HHP) on the binding of ANS to BSA in the presence of the three salts and in the temperature range between 5 and 25 °C. To this end, we performed the same titration experiments following the complex formation by means of HHP fluorescence spectroscopy. As an example, Figure 4 depicts the binding isotherms obtained for the ANS titration with a solution of BSA in the presence of 250 mM MgCl_2_ at *T* = 25 °C and in the pressure range between 1 and 2000 bar. The binding constants determined for all samples are shown in Table 2.

An inspection of Table 2 reveals that pressure has an effect on the values of *K*_b_ that depends on the temperature and on the type of salt present in solution. It is important to note that BSA is stable in the whole pressure range covered and no unfolding and denaturation of the protein takes place [25,26,27]. Thus, variations observed in the values of *K*_b_ can most likely not be ascribed to protein conformational changes imposed by pressure. In the presence of MgCl_2_, at 25 °C, only minor effects on the binding affinity were observed. The same holds true for *T* = 15 °C. Instead, the pressure effect seems more pronounced at *T* = 5 °C, were a clear increase of *K*_b_ was observed upon a pressure increase from 1 to 2000 bar. Thus, the application of pressure favors the formation of the complex at lower temperatures.

Instead, in the MgSO_4_ containing buffer, at 25 °C, a slight decrease of the affinity is detected upon pressurization. The volume changes observed are very small (for comparison, the molar volume of one water molecule is about 18 mL mol^−^^1^). The pressure effect seems to be slightly favorable for binding at 15 °C. At *T* = 5 °C, *K*_b_ decreases slightly with increasing pressure. Thus, depending on the temperature, the complex formation in the presence of MgSO_4_ is slightly favored or disfavored. The changes are quite small or even negligible, however.

In the presence of Mg(ClO_4_)_2_, at *T* = 25 °C, the first binding constant of the two remaining binding sites, *K*_b1_, decreases slightly with increasing pressure. Instead, the second binding mode, *K*_b2_, does not seem to be significantly affected by pressure changes up to the 2000 bar regime. At 15 °C, *K*_b1_ and *K*_b2_ increase slightly upon pressurization. Moreover, at the lowest temperature, at *T* = 5 °C, *K*_b1_ and *K*_b2_ increase with increasing pressure. Thus, the two binding modes respond to pressure in different ways, highlighting that the packing properties and hydration effects upon binding, which largely contribute to volume changes, are different for each binding site of the protein.

From the pressure dependence of the *K*_b_ values, the volume change upon binding, the protein–ligand binding volume Δ*V*_b_, can be determined (for an in-depth discussion of pressure effects on ligand binding, please refer to Reference [28] and references therein):

(1) $${\left[\frac{\partial \ln K_{b}}{\partial p}\right]}_{T}=-\frac{\Delta V_{b}}{RT}$$

**Figure 5:**
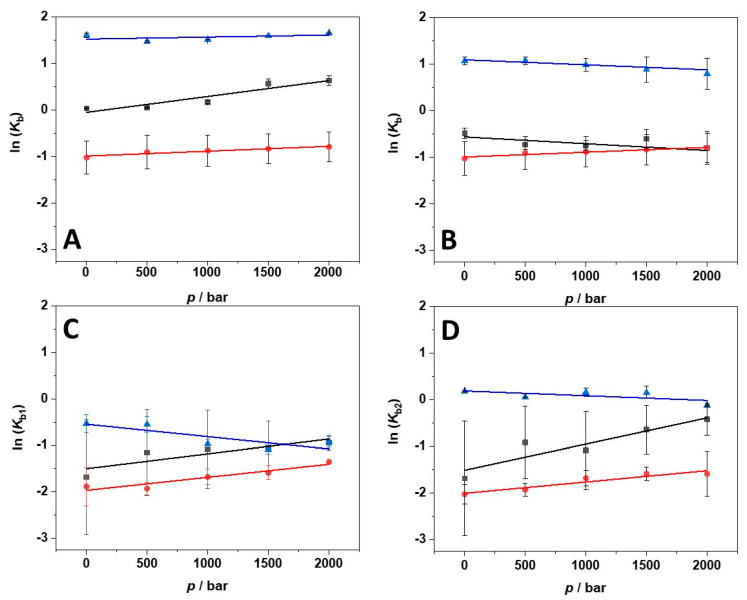
Pressure dependence of the binding constant (*K*_b_) for the complex formation between BSA and ANS in the presence of 250 mM MgCl_2_ (**A**), MgSO_4_ (**B**), and Mg(ClO_4_)_2_ (**C**) and (**D**) at the temperatures of 5 °C (black squares), 15 °C (red circles), and 25 °C (blue triangles). The plot in (**C**) refers to the pressure dependence of the binding constant of the first binding site (*K*_b1_), the plot in (**D**) to that of the second binding site (*K*_b2_). From the slopes of ln(*K*_b_) vs. *p*, the binding volume (Δ*V*_b_) can be calculated by means of Equation (1).

Here, [∂ln*K*_b_/∂*p*]*_T_* is the derivative of the logarithm of the binding constant (*K*_b_) with respect to pressure (*p*) at constant temperature (*T*). *R* is the universal gas constant. Δ*V*_b_ is the binding volume defined as the difference between the partial molar volume of the complex (PL, where P is the protein and L the ligand) and the sum of the partial molar volumes of the uncomplexed state (P+L). Thus, the binding volume can be directly estimated by taking the slope of a plot of ln*K*_b_ vs. *p*, assuming that the volume change is independent of pressure in the (here rather small) pressure range covered. Figure 5A–D depict the plots of ln*K*_b_ vs. *p* obtained in the salt-containing media and at the three temperatures measured. All Δ*V*_b_ values obtained are collected in Table 3.

**Figure 6:**
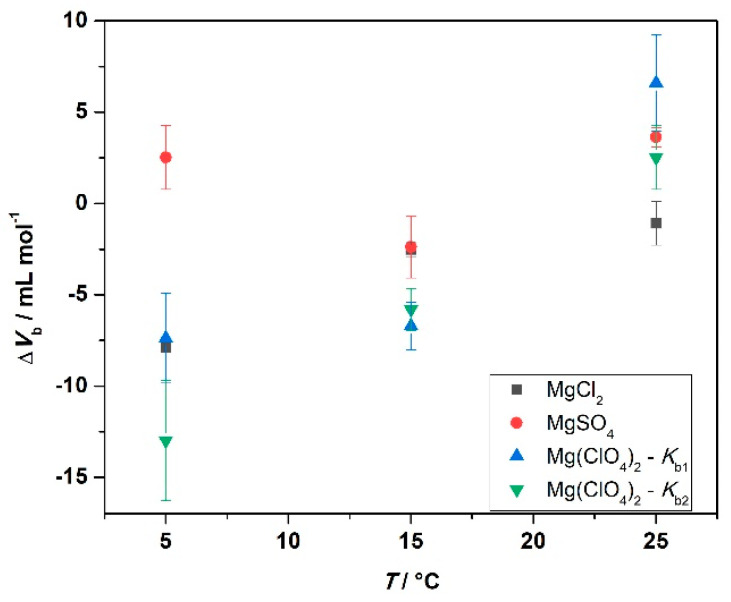
Plot of binding volumes, Δ*V*_b_, as a function of temperature for the indicated 250 mM salt solutions.

At *T* = 25 °C, the binding volume in the presence of MgCl_2_ is close to zero, highlighting the negligible effect of pressure on the complex formation. Instead, in the sulfate-containing solution, the volume change is slightly positive, i.e., the application of pressure does not favor the interaction between the protein and the ligand. In the presence of perchlorate, the binding volumes of both binding sites are positive. However, Δ*V*_b1_ > Δ*V*_b2_, indicating that the two binding sites are characterized by different packing and hydration properties. In the presence of MgCl_2_, the decrease of temperature is accompanied by more negative Δ*V*_b_ values; this is to say, decreasing temperature and increasing pressure both favor the complex formation. Instead, in the MgSO_4_-containing buffer, Δ*V*_b_-changes with temperature are less pronounced. A small decrease of Δ*V*_b_ was observed, only. Interestingly, in the presence of Mg(ClO_4_)_2_, the temperature had a comparably strong impact on the volume changes. Both binding volumes become negative, reaching values of −7.4 and −12.9 mL mol^−^^1^ for the first and the second binding site, respectively. Thus, when magnesium perchlorate is present in solution, the combined increase of pressure and decrease of temperature strongly favor the complex formation. To highlight the changes of binding volume with temperature, Δ*V*_b_ (*T*) is shown for the different salts in Figure 6.

## 4. Discussion

In this work, the combined effects of temperature and pressure on the binding characteristics of ANS to BSA were determined in the presence of Mars-relevant salts, including MgCl_2_, Mg(ClO_4_)_2_, and MgSO_4_. We found that the binding of the small aromatic ligand ANS to BSA depends significantly on the type of salt, i.e., the anion, on temperature, and on pressure. At ambient conditions (*T* = 25 °C and *p* = 1 bar), the presence of 250 mM MgCl_2_ and MgSO_4_ has no impact on the stoichiometry and no major effect on the binding strength of the complex formed. Both the binding stoichiometry and *K*_b_ are similar to those previously determined in neat buffer solution (three ANS molecules are bound to one BSA with a *K*_b_ = 4.2∙10^6^ M^−^^1^ at 25 °C) [17].

Conversely, the presence of perchlorate perturbs the binding characteristics of ANS to BSA. Only two ANS molecules are now bound to BSA, and the two binding sites are characterized by different binding constants, i.e., the equivalence of binding sites is lost. This phenomenon could be due to the direct interaction of the perchlorate anion with BSA. Indeed, an interaction of perchlorate ions with charged residues of lysozyme was previously reported [29]. The interaction with BSA of other anions of such low charge density (e.g., iodide) was also reported [30]. Furthermore, owing to its low charge density, perchlorate has almost hydrophobic characteristics and can establish interactions with hydrophobic patches of proteins as well.

BSA is a heart-shaped protein of about 66 kDa with high homology with the human counterpart, HSA [24,31]. Its structure is composed by three domains, each of which is composed of two subdomains. A docking study performed on this system identified two potential binding sites for ANS in the subdomain IIIA and another one in the subdomain IB [17]. The two sites in the subdomain IIIA are buried in the inner core of the protein. Conversely, the other site is localized in the more solvent-exposed subdomain IB. Thus, it is possible that perchlorate can easily get access to the site in the subdomain IB, thereby completely hampering the binding of ANS to this site. The binding of perchlorate can occur through both hydrophobic and electrostatic interactions. Indeed, an inspection of the crystal structure revealed the presence of several positively charged residues (Lys, Arg, and His) that could establish interactions with anions such as perchlorate. Instead, the two sites in the subdomain IIIA are buried in the inner core of the protein. In this subdomain, some positively charged residues are also present. The loss of equivalence between the two different sites can most likely be ascribed to the interaction of perchlorate with one of the sites that, in this case, can partially occlude it, which leads to the observed decrease in the binding affinity (*K*_b1_ = 5.9∙10^5^ M^−1^, *K*_b2_ = 1.2∙10^6^ M^−1^ at *T* = 25 °C).

A decrease of temperature, at ambient pressure, leads to an overall decrease of the binding constant of the ligand. Such a decrease could be due to an enhancement of the transient interactions of the salt anions and the protein in the binding area, and possibly also of the Mg^2+^ cation with the ligand, which decreases the activity (coefficient) of ANS. Indeed, the binding of anions such as chloride and perchlorate to carbonic anhydrase II, for example, is characterized by negative enthalpy changes [32], pointing out that a decrease of temperature should favor their binding process. It is important to recall that the binding of ANS to proteins is mainly but not exclusively due to hydrophobic interactions. The negatively charged sulfate moiety of ANS is able to establish electrostatic interactions with positively charged residues of the protein [33,34,35]. Consequentially, the presence of bound anions can partially affect the binding of ANS, leading to a reduction of the *K*_b_-values with decreasing temperature. From the temperature dependence of *K*_b_, it is possible to determine the enthalpy change for the binding process by means of the van’t Hoff relation, assuming that the enthalpy change is independent of temperature [36]. Here, we found that the ln*K*_b_ vs. 1*/T* plots are non-linear for all solution conditions studied (data not shown). The non-linearity could be ascribed to the complexity of the systems where more than one ANS molecule is bound to BSA, with the binding sites having different binding enthalpies owing to their different chemical makeup. Further, competitive binding by salt anions such as SO_4_^2−^ and ClO_4_^−^ will be temperature-dependent as well.

Small but significant effects of pressure were observed for the binding process of ANS to BSA, with the effect being dependent both on the temperature and on the nature of the salt anion. Inspection of Table 3 and Figure 6 discloses several interesting points. In the presence of MgCl_2_, the binding volume, Δ*V*_b_, is close to zero at 25 °C; that is, there is no pressure effect on the complex formation at ambient temperature (up to 2 kbar). However, at lower temperatures, pressure favors the interaction, reaching a Δ*V*_b_ value of about −8 mL mol^−1^ at *T* = 5 °C, which translates to an increase of *K*_b_ by about a factor of two. A similar behavior was observed in the presence of Mg(ClO_4_)_2_, for which a negative Δ*V*_b_ was found for both binding sites at 5 °C. Negative Δ*V*_b_-values imply that the partial molar volume of the complexed state (*V*_PL_) is smaller than that the partial molar volumes of the uncomplexed state (*V*_P_ + *V*_L_). A possible reason for such a negative binding volume could be dense packing and a decrease of void volume in the binding site upon ligand binding, which might be expected to be more pronounced at low temperatures, where the dynamics of the protein’s surface groups is expected to be reduced. Upon ligand binding, in an induced-fit kind of scenario, tightening of the internal atomic packing of the protein molecule might take place, which leads to a decrease of the volume fluctuations of the protein and hence a decrease of the partial molar volume of the protein. A possible origin for a positive Δ*V*_b_-value is most likely a change in the hydration of the protein binding site and/or the ligand(s). As hydration water of proteins generally has a slightly higher density compared to bulk water [37], dehydration results in a small volume increase of the system, with the effect being more pronounced at low temperature. In the presence of solutions of high ionic strength, where large electrostrictive effects prevail in bulk solution, in particular in the presence of small cations and anions of high charge density, such an effect might become less pronounced or even reversed, however. Positive Δ*V*_b_-values could also be due to creation of void volume upon ligand binding if, for example, the binding site is partially obstructed by salt ions, which might be the case for the sulfate anion at low temperatures. Further, the strength of the binding of the anions to the protein might also be pressure sensitive. Application of high pressure could disfavor the binding of the salt anions to the proteins by weakening electrostatic interactions, which would allow an enhancement of the interaction between the ligand ANS and BSA, leading to an increase of *K*_b_ [38,39]. The sulfate and perchlorate data do not seem to support this idea, however.

## 5. Conclusions

In summary, we have found that the binding of a small aromatic ligand such as ANS to an archetypical multifunctional protein, BSA, depends not only significantly on temperature, but also on the type of salt and, to a lesser extent, on pressure. A significant decrease in the binding constant is observed at ambient conditions only in the presence of high concentrations the perchlorate. Interestingly, an increase in pressure and a decrease in temperature favor the binding process of the ligand, rendering the binding volume more negative.

Aside from advancing our general understanding of how ions influence protein–ligand interactions under non-standard temperatures and pressures, our work was specifically focused on ions found on Mars. Our motivation was to understand whether the more water-rich past of that planet, and the potentially more liquid, water-rich subsurface of the planet even today, hosts conditions theoretically conducive to life. In other words, do conditions such as high pressure and the presence of perchlorate ions render a planet uninhabitable with respect to known biochemistry? As our data show, chloride and sulfate ions do not significantly alter protein–ligand interactions under a range of temperature and pressure conditions. However, the chaotropic perchlorate ion does influence protein–ligand binding more profoundly, but it does not abolish it, implying that with respect to temperature, pressure and the presence of perchlorate ions, Martian conditions do not necessarily exclude protein–ligand interactions of the type examined here, but that modifications in binding sites could be required for life to theoretically persist under such geochemical conditions. We note that our data also imply that protein–ligand interactions would not necessarily be substantially altered in the high concentrations of sulfate ions associated with crustal fluids in the terrestrial deep subsurface, providing new insights into the environment for deep subsurface biochemistry on our own planet.

## Figures and Tables

**Table 1:** Binding constants (*K*_b_) and stoichiometries for the BSA:ANS complex formation in 10 mM Tris-HCl buffer, pH 7.4, in the presence of the indicated salts and at temperatures (*T*) of 5, 15, and 25 °C.

Solution Conditions	*T*/°C	*K*_b1_/M^−1^ × 10^6^	*K*_b2_/M^−1^ × 10^6^	^1^ *n*
250 mM MgCl_2_	5	1.0 ± 0.1	-	1:3
	15	0.36 ± 0.13	-	1:3
	25	4.9 ± 0.2	-	1:3
250 mM MgSO_4_	5	0.62 ± 0.15	-	1:3
	15	0.39 ± 0.14	-	1:3
	25	2.9 ± 0.3	-	1:3
250 mM Mg(ClO_4_)_2_	5	0.19± 0.13	0.19 ± 0.13	1:2
	15	0.15 ± 0.04	0.13 ± 0.02	1:2
	25	0.59 ± 0.22	1.2 ± 0.1	1:2

Note: ^1^*n* represents the stoichiometry of binding as BSA:ANS.

**Table 2:** Binding constants (*K*_b_) and stoichiometries (*n*) for the BSA:ANS complex formation in 10 mM Tris-HCl buffer, pH 7.4, in the presence of the indicated salts, temperatures, and pressures.

Solution Conditions	*T*/°C	*p*/bar	*K*_b1_/M^−1^ ∙10^6^	*K*_b2_/M^−1^ ∙10^6^	^1^ *n*
250 mM MgCl_2_	5	1	1.0 ± 0.1	-	1:3
	5	500	1.0 ± 0.2	-	1:3
	5	1000	1.2 ± 0.3	-	1:3
	5	1500	1.8 ± 0.3	-	1:3
	5	2000	1.9 ± 0.3	-	1:3
250 mM MgCl_2_	15	1	0.36 ± 0.13	-	1:3
	15	500	0.40 ± 0.16	-	1:3
	15	1000	0.41 ± 0.16	-	1:3
	15	1500	0.43 ± 0.17	-	1:3
	15	2000	0.45 ± 0.18	-	1:3
250 mM MgCl_2_	25	1	4.9 ± 0.2	-	1:3
	25	500	4.3 ± 0.1	-	1:3
	25	1000	4.5 ± 0.1	-	1:3
	25	1500	4.9 ± 0.2	-	1:3
	25	2000	5.2 ± 0.1	-	1:3
	5	1	0.62 ± 0.15	-	1:3
	5	500	0.48 ± 0.07	-	1:3
250 mM MgSO_4_	5	1000	0.47 ± 0.07	-	1:3
	5	1500	0.55 ± 0.20	-	1:3
	5	2000	0.44 ± 0.20	-	1:3
	15	1	0.39 ± 0.14	-	1:3
	15	500	0.40 ± 0.16	-	1:3
250 mM MgSO_4_	15	1000	0.42 ± 0.15	-	1:3
	15	1500	0.43 ± 0.17	-	1:3
	15	2000	0.45 ± 0.18	-	1:3
	25	1	2.9 ± 0.3	-	1:3
	25	500	2.9 ± 0.3	-	1:3
250 mM MgSO_4_	25	1000	2.7 ± 0.4	-	1:3
	25	1500	2.4 ± 0.7	-	1:3
	25	2000	2.2 ± 0.9	-	1:3
	5	1	0.19± 0.13	0.19 ± 0.13	1:2
	5	500	0.32 ± 0.25	0.40 ± 0.33	1:2
250 mM Mg(ClO_4_)_2_	5	1000	0.34 ± 0.26	0.34 ± 0.26	1:2
	5	1500	0.35 ± 0.19	0.52 ± 0.43	1:2
	5	2000	0.39 ± 0.07	0.66 ± 0.53	1:2
	15	1	0.15± 0.04	0.13 ± 0.13	1:2
	15	500	0.14 ± 0.01	0.15 ± 0.10	1:2
250 mM Mg(ClO_4_)_2_	15	1000	0.19 ± 0.02	0.19 ± 0.18	1:2
	15	1500	0.21 ± 0.02	0.21 ± 0.21	1:2
	15	2000	0.26 ± 0.01	0.24 ± 0.20	1:2
	25	1	0.59 ± 0.22	1.2 ± 0.1	1:2
	25	500	0.59 ± 0.17	1.1 ± 0.1	1:2
250 mM Mg(ClO_4_)_2_	25	1000	0.38 ± 0.10	1.2 ± 0.2	1:2
	25	1500	0.34 ± 0.03	1.2 ± 0.3	1:2
	25	2000	0.40 ± 0.05	0.89 ± 0.05	1:2

Note: ^1^*n* represents the stoichiometry of binding as BSA:ANS.

**Table 3:** Binding volumes (Δ*V*_b_) for the BSA:ANS complex formation at the indicated temperatures and in the presence of 250 mM MgCl_2_, MgSO_4_, and Mg(ClO_4_)_2_. The Δ*V*_b_ values are reported in mL mol^−1^ of protein.

	MgCl_2_	MgSO_4_	Mg(ClO_4_)_2_	Mg(ClO_4_)_2_
*T*/°C	Δ*V*_b_/mL mol^−1^	Δ*V*_b_/mL mol^−1^	Δ*V*_b1_/mL mol^−1^	Δ*V*_b2_/mL mol^−1^
5	−7.9 ± 0.1	2.0 ± 1.0	−7.4 ± 2.4	−12.9 ± 3.2
15	−2.5 ± 0.4	−2.0 ± 1.0	−6.0 ± 1.0	−5.0 ± 1.0
25	−1.0 ± 1.0	3.6 ± 0.5	6.0 ± 2.0	2.0 ± 1.0

## Data Availability

The data presented in this study are available on request from the corresponding authors.

## Supplementary Materials

The following are available online at https://www.mdpi.com/article/10.3390/biology10070687/s1. Figure S1: Job’s plot for ANS:BSA complex formation obtained in the presence of 250 mM MgCl_2_ at the temperature of (A) 5 °C and (B) 15 °C and at the pressure of 1 bar. The total concentration ([ANS] + [BSA]) was 35 µM. Figure S2: Job’s plot for ANS:BSA complex formation obtained in the presence of 250 mM MgSO_4_ at the temperature of (A) 5 °C and (B) 15 °C and at the pressure of 1 bar. The total concentration ([ANS] + [BSA]) was 35 µM. Figure S3: Job’s plot for ANS:BSA complex formation obtained in the presence of 250 mM Mg(ClO_4_)_2_ at the temperature of (A) 5 °C and (B) 15 °C and at the pressure of 1 bar. The total concentration ([ANS] + [BSA]) was 35 µM. Figure S4: Far-UV CD spectra of BSA at the temperature of (A) 5 °C and (B) 15 °C, in the presence of 250 mM MgCl_2_ (red spectrum), MgSO_4_ (blue spectrum), and Mg(ClO_4_)_2_ (green spectrum). For reference, the CD spectrum of BSA in neat buffer condition (black spectrum) is also reported. All the spectra were acquired in 10 mM Tris-HCl buffer, pH 7.4, using a 0.01 cm path-length quartz cuvette.
