# Supplementary material for: The Effects of Temperature and Pressure on Protein-Ligand Binding in the Presence of Mars-Relevant Salts
Source: Biology (Basel). 2021 Jul 20;10(7):687. doi: 10.3390/biology10070687 (PMC8301423; doi:10.3390/biology10070687)
Supplement: Supplementary file 1 [file biology-10-00687-s001.zip › biology-1293633-supplementary.pdf]

Supplementary Materials

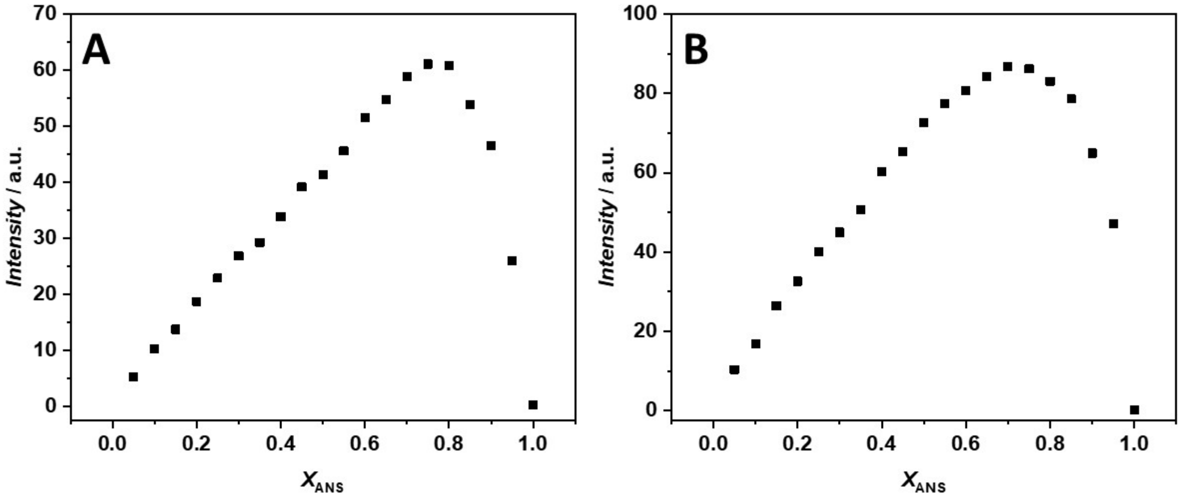

Figure S1. Job's plot for ANS:BSA complex formation obtained in the presence of 250 mM $MgCl_2$ at the temperature of (**A**) 5 °C and (**B**) 15 °C and at the pressure of 1 bar. The total concentration ((ANS) + (BSA)) was 35 μM.

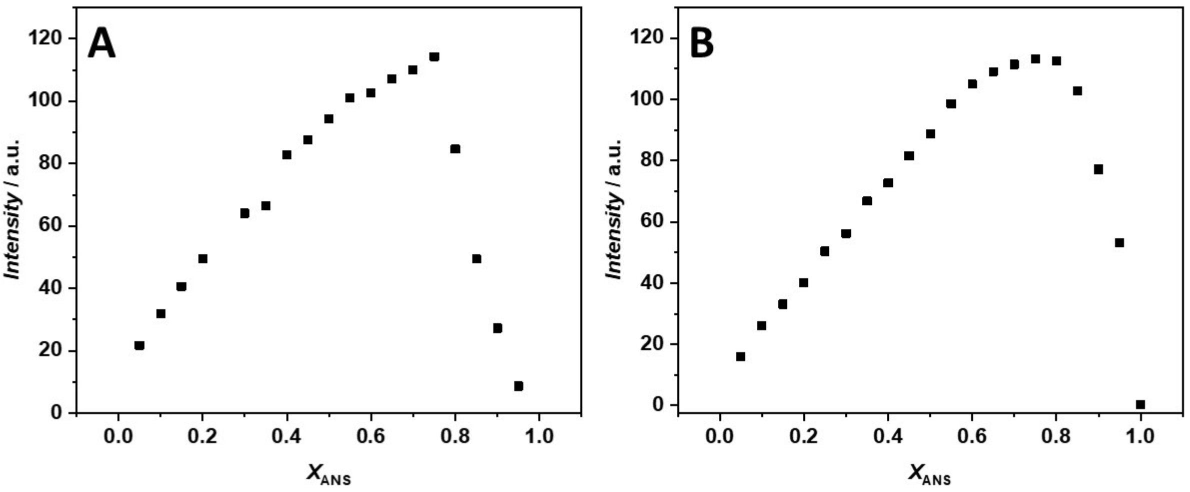

Figure S2. Job's plot for ANS:BSA complex formation obtained in the presence of 250 mM $MgSO_4$ at the temperature of (**A**) 5 °C and (**B**) 15 °C and at the pressure of 1 bar. The total concentration ((ANS) + (BSA)) was 35 μM.

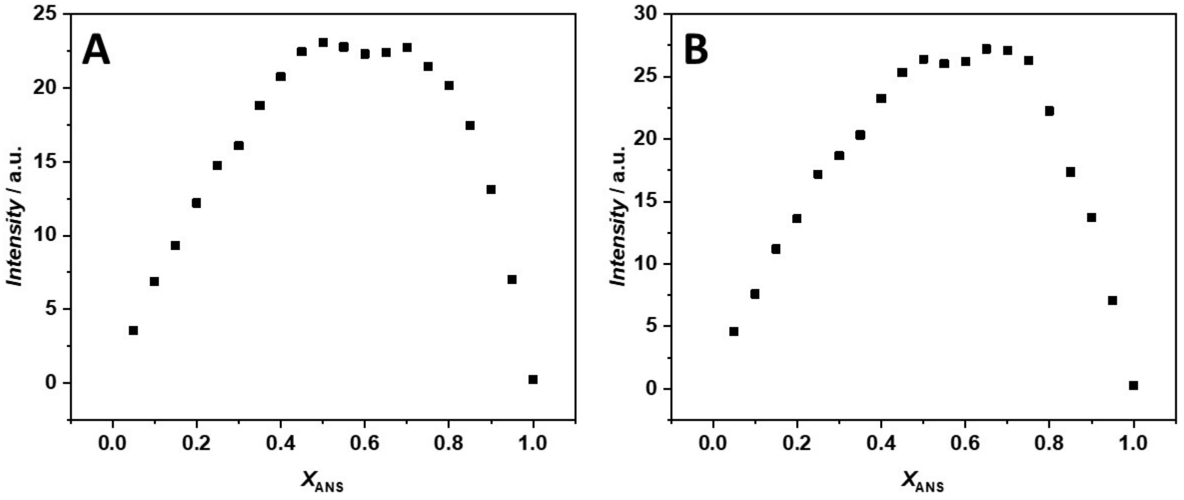

Figure S3. Job's plot for ANS:BSA complex formation obtained in the presence of 250 mM $Mg(ClO_4)_2$ at (**A**) 5 °C and (**B**) 15 °C and at the pressure of 1 bar. The total concentration ((ANS) + (BSA)) was 35 μM.

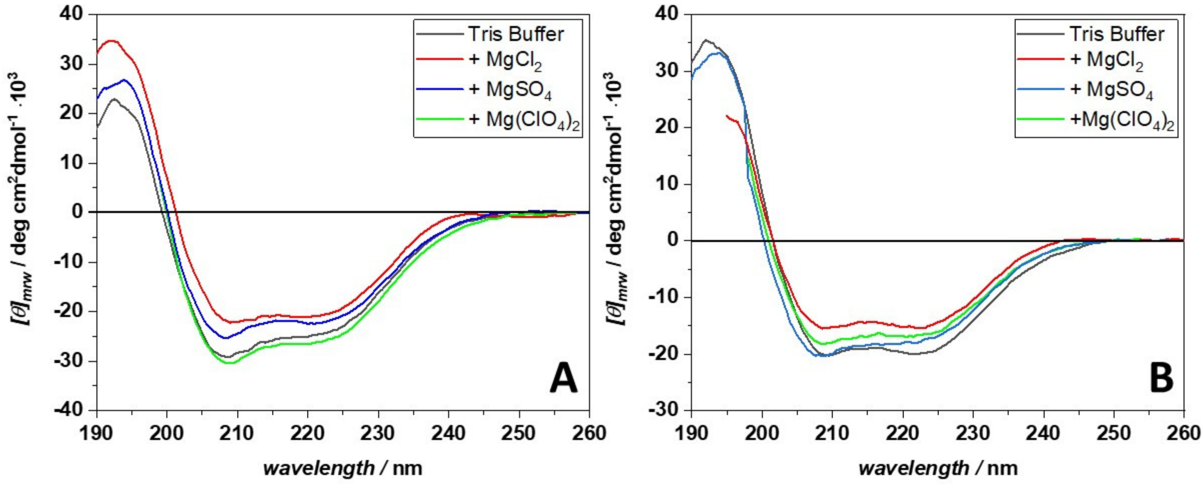

Figure S4. Far-UV CD spectra of BSA at the temperature of (**A**) 5 °C and (**B**) 15 °C, in the presence of 250 mM $MgCl_2$ (red spectrum), $MgSO_4$ (blue spectrum), and $Mg(ClO_4)_2$ (green spectrum). For reference, the CD spectrum of BSA in neat buffer condition (black spectrum) is also reported. All the spectra were acquired in 10 mM Tris-HCl buffer, pH 7.4, using a 0.01 cm path-length quartz cuvette.
